# StegoFrameOrder—MAC Layer Covert Network Channel for Wireless IEEE 802.11 Networks

**DOI:** 10.3390/s21186268

**Published:** 2021-09-18

**Authors:** Krzysztof Sawicki, Grzegorz Bieszczad, Zbigniew Piotrowski

**Affiliations:** 1 Institute of Optoelectronics, Military University of Technology, 00-908 Warsaw, Poland; grzegorz.bieszczad@wat.edu.pl; 2 Faculty of Electronics, Military University of Technology, 00-908 Warsaw, Poland; zbigniew.piotrowski@wat.edu.pl

**Keywords:** network security, wireless networks, covert channel, steganography, protocol-trojan

## Abstract

The proposed StegoFrameOrder (SFO) method enables the transmission of covert data in wireless computer networks exploiting non-deterministic algorithms of medium access (such as the distributed coordination function), especially in IEEE 802.11 networks. Such a covert channel enables the possibility of leaking crucial information outside secured network in a manner that is difficult to detect. The SFO method embeds hidden bits of information in the relative order of frames transmitted by wireless terminals operating on the same radio channel. The paper presents an idea of this covert channel, its implementation, and possible variants. The paper also discusses implementing the SFO method in a real environment and the experiments performed in the real-world scenario.

## 1. Introduction

Wireless networks are extensively used nowadays in personal, commercial, or small office applications and professional or critical infrastructure such as security cameras and sensors, industrial automation, autonomous drone management, etc. For this reason, the wireless network security is a concern of the scientific and industrial community but mainly focuses on the security of the used protocols, cryptographic strength, and implementation flaws. Cryptography is not the only method to secure data transmission in wireless networks. A different approach is to use steganography—a technique that enables the data transmission in a hidden manner. A definition of steganography was provided in [1,2], but in this paper we restrict it to network steganography [3]—steganographic methods exploiting computer networks. The critical factor for steganography is to make the data ’covert’ so that no one would be able to receive the information. This is the fundamental difference between cryptography and steganography. Using cryptography, the data can be received by virtually anyone but cannot be decrypted by anyone except the authorised recipient. Steganography may be treated as complementary to cryptography and is an alternative approach that is (by its definition) rarely reported to be used as its security is related to algorithm secrecy.

Steganographic methods create covert channels—the type of communication channels where the data are transmitted with the use of steganographic method. The element that must be kept secret is the method of hiding data in the covert channel. It is similar to the encryption key in cryptography which is also a secret for the third side.

Covert channels apply when the fact of the presence of the communication should be kept secret. For example, when the devices are placed in the enemy’s territory, when the data must be exchanged in public place but without direct contact between devices, or when commonly used devices have a dual purpose and the secondary functionality is meant to be secret.

A serious concern for some of the adopters of telecommunication technologies is an intentional implementation of security flaws—software and hardware Trojans [4]. Intentional security flaws can be used to provide a backdoor to the system or to leak crucial security information (for example, cryptographic keys). For such an application, even a very low bandwidth provided by covert channels can be a severe security threat [5,6]. Sometimes, an intentionally introduced hidden undocumented protocol is provided for valuable purposes such as emergency control protocol, covert authentication, and cryptographic key exchange. The wide range of applications of the steganography (command and control communication for malware, hiding malicious code, exfiltrating user data, etc.) has been described in detail by K. Cabaj et al. [7].

The article presents a concept and implementation of new steganographic method named StegoFrameOrder (SFO). The method exploits a race condition between frames sent from two or more wireless stations to send covert data. The presented implementation is based on modification of the Distributed Coordination Function (DCF) found in IEEE 802.11 wireless networks. However, the general concept can be used in any network protocol using a non-deterministic medium access algorithm. The method embeds hidden bits of information in the relative order of frames transmitted by at least two wireless terminals operating on the same radio channel.

The proposed method of hiding data in IEEE 802.11 wireless networks involves the cooperation of the chosen subset of connected devices (computers, smartphones, and different types of sensors) within the network. In order to extract hidden data, a listening network node is introduced that senses the traffic in the wireless network but does not necessarily need to be an active part of the network. This feature is critical as the information leakage can be carried out even to the node not authenticated in a victim Wi-Fi network.

## 2. Related Work

Network steganography, in general, becomes an important topic in the network security area [8,9,10], and many network steganography methods have been proposed in the literature.

Steganography can be considered as a security risk [7] or as security support [11,12]. Network security systems, especially in the area of Internet of Things devices, despite the rapid development [13,14,15] and the use of many modern and innovative techniques [16,17] are often not prepared for the possible use of covert channels. The occurrences of such cases have been already reported by specialized companies [18,19].

### 2.1. Covert Channel Types

One can distinguish between two types of covert channels (communication channels created by steganographic methods) in computer networks: timing channels and storage channels [20]. Some steganographic methods require the receiver to be connected to the network. However, others allow for the receipt of hidden data by a designated device (the sensor) that is not connected to the given wireless network and only listens to the transmitted data. This model of passive steganography is used both in network [21] and acoustic steganography [22].

#### 2.1.1. Storage Covert Channels

In network steganography, in the storage channel class, the covert channel is most often created by altering the header fields or exploiting the available padding fields used by various network protocols. The basic methods use, for example, the Padding and Type of Service fields in the IPv4 header or the Reserved field in the TCP header. The example of this kind of covert channels has been presented in [23]. A very interesting and simple idea was proposed in [24,25]: the use of the *Identification* field in the IPv4 header. There are also known solutions based on creating customised, non-standard types of packets or frames to transmit covert data. One of such approach was proposed in [26].

In some cases, the header fields cannot be freely altered because they may break or disturb regular (non-covert) network traffic. Since covert data are embedded in header fields used by the protocols, one needs to be sure that the network protocol works correctly even after modifying the header fields. The actions taken to ensure this often limits the bandwidth of the covert channel as the usage rules of the header fields are strictly defined. One example method of using header field was described in [27,28], and it used the *Time to Live* field on the IPv4 header to transmit additional covert data. The *TTL* field, which is eight bits long, can be altered, and one byte of covert data can be embedded into it. However, because of the principle of operation of the IP protocol, small values of *TTL* field cannot be used. This constraint means that practically only five or six bits in the *TTL* field can be used for covert transmission, with regular IP transmission still operational. Another interesting method, which can be applied in wireless networks, is the altering of the *Timestamp* field in Beacon frames broadcasted by IEEE 802.11 access points [29]. This method modifies the least significant bits of that field to embed covert data. This kind of modification does not affect the operation of the wireless network. Moreover, the receiver of this covert data can be completely independent of the network and only needs to listen to and intercept the beacon frames. A model example of using altered *Sequence No.* and *Fragment No.* fields of the IEEE 802.11 frame in a practical application was described in [30].

Network steganography is often implemented in transport layer protocols. In the TCP protocol, the *Sequence Number* (numerical value of the initial sequence) field can be used for covert transmission [24] to send a maximum number of 16 bits of covert data for one TCP session. This solution complicates the use of the protocol, but it is an excellent example of a transport layer steganography. Other transport layer protocols, such as UDP, can also be subjects of network steganography. Such a method has been presented in [31]—the covert data is embedded in the length of UDP datagram—and authors claim to achieve 456 $\frac{b}{s}$ of bandwidth of the covert channel established with that method.

In some cases, it is possible to transmit covert data by altering the header fields used and intentionally disturbing protocol communication. Paper [32] presents the example of exploiting this approach. The HICCUPS method involves embedding the covert data into IEEE 802.11 frames with incorrect checksum (*FCS*) values. A set of similar methods has been described in [33].

#### 2.1.2. Timing Covert Channels

Timing methods are more complicated than the methods presented above. In general, they exploit different parameters of timing relations during data transmission. The typical timing method has been described in [34]. Covert data are transmitted by selecting an appropriate value of the backoff time that is usually used to enable collision avoidance in medium access protocols. The backoff time is randomised for every frame in the Wi-Fi network. Thus, by deliberately increasing or reducing the backoff time (the delay in frame transmission start), it is possible to create a covert channel. An interesting description of these methods can be found in [35]. Typical timing solutions for IEEE 802.11 networks that take advantage of their properties (e.g., non-deterministic media access) are also available. An example of such a method has been described in [36]. Another solution for the timing covert channel has been presented in [37]. This covert channel was implemented by scanning TCP ports in a specific order.

The paper proposes a new and original data hiding method named StegoFrameOrder (SFO) that can be classified as a timing method. The SFO operates in the second layer of the ISO/OSI reference model (link layer). The functions controlling its operation are implemented in the third layer of the ISO/OSI reference model (the network layer). The method needs customised interlayer communication mechanisms to operate. Data reception is implemented by an RF-passive wireless receiver that does not need to implement more than the first and the second layers of the ISO/OSI reference model.

## 3. Description of Proposed Method

### 3.1. DCF and CSMA/CA

**Figure 1:**
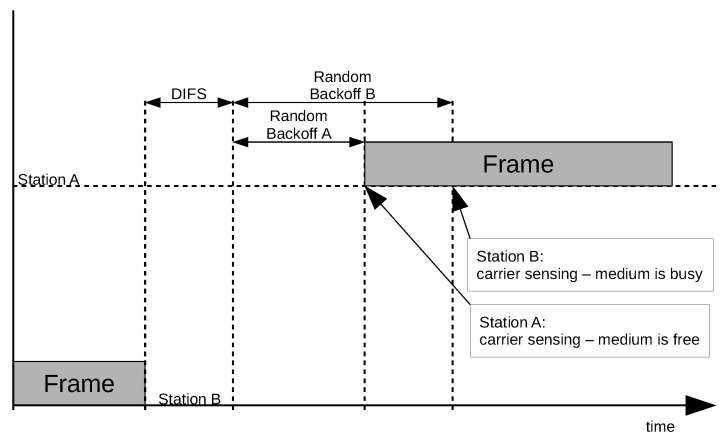
Example of medium access control in IEEE 802.11 networks.

The distributed coordination function (DCF) and the carrier-sense multiple access/collision avoidance (CSMA/CA) algorithms are used in IEEE 802.11 wireless networks for medium access control. Before media access, every station has to determine whether the medium is busy. The DCF and CSMA/CA operation principles are based on randomisation of the period during which the station awaits for media access (the backoff time). Randomisation of this time significantly reduces collision probability. The scenario where two stations try to start transmitting is shown in Figure 1.

The station that wants to start transmission of a frame waits for the distributed interframe space (DIFS) period, counted from the end of the transmission of the previous frame, and then randomises the backoff time. A station that has already waited for its calculated backoff time (station A in Figure 1) checks whether the medium is busy (another frame transmission takes place). If the medium is not busy, the station starts the transmission. Meanwhile, station B, which has calculated a longer backoff time, checks if the medium is busy at the end of the waiting period. However, as another frame (originating from station A) is being transmitted, the process is postponed until the end of the ongoing transmission.

The duration of the backoff time is randomised by using a specific interval. The length of this interval depends on the standard used by the stations or the type of transmission they perform (e.g., voice, video, or regular transmission). Moreover, it is possible to modify the random value of the backoff time by the appropriate customisation of the wireless card drivers. If the wireless network stations use the exact value of the backoff randomisation interval, they have an equal chance of transmitting frames, and none of them are prioritised.

### 3.2. Using Relative Order of Frames to Transmit Covert Information

**Figure 2:**
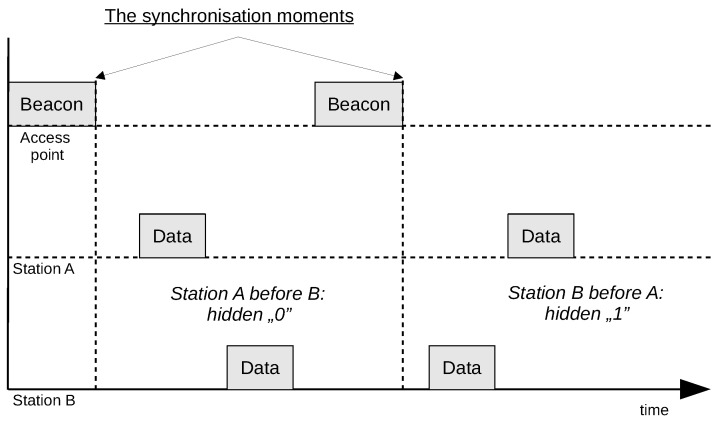
Example of covert bit transmission.

Transmitting covert information by using the relative order of frames embeds information in the timing relation of frames being sent by multiple participating terminals. Its operation requires at least two wireless network terminals that send data frames of regular, non-covert information. The covert information bits are encoded in two cases: Station A transmits the frame before station B (e.g., bit 0), and station B transmits the frame before station A (e.g., bit 1). Covert information is conveyed by order of the frames transmitted from the stations to the access point (with the *ToDS* field set to one). Frames that are retransmitted by the access point (with the *FromDS* field set to one) are not considered. It is also necessary to designate the moment at which the relative frames order check begins (the synchronisation moment). Such time may be, for example, the end of a beacon frame transmitted by the access point. An example of covert bit transmission is shown in Figure 2. It does not show frames retransmitted by the access point as they are not relevant in the proposed method.

The required order of data frames is obtainable if at least one station deliberately delays or speeds up its transmission (e.g., by modifying the backoff time to speed up the transmission or holding the frame in a buffer for additional transmission delay). The method involves embedding covert information in the mutual order of frames that first follows the beacon frame. If two or more frames are sent from the same station between successive beacon frames, only the first one is considered.

The covert data can be received by any station that can receive frames from the access point as well as from both stations. The receiving station needs to be able to identify the stations participating in hidden transmission, the coding scheme of the covert data, and the method of synchronisation used. It does not need to be associated with the wireless network or to know the encryption key, as the overt data transmitted by the network are not essential for this method to work.

### 3.3. Transmission Synchronisation

The method demands some sort of synchronisation mechanism—a moment when the relative order of frames is considered. Without the synchronisation mechanism, there would be a need for unrealistic network constraints such as an exactly equal count of frames sent from two stations or ideally synchronised real-time clocks in participating nodes. A synchronisation mechanism can provide a moment in time from which a race between frames can start.

**Figure 3:**
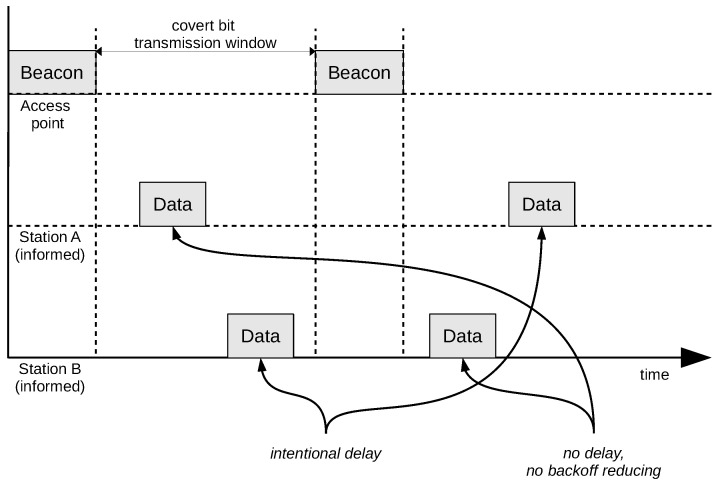
Both-informed variant of the SFO method.

Synchronisation can be carried out by using the beacon frames that are periodically sent by the access point. In the presented method, the moment at which the transmission of the beacon frame ends is treated as the beginning of the transmission window for the covert bit (Figure 3). An occurrence of synchronisation signal allows for the transmission of a single bit of covert information. To send longer messages, one must use multiple synchronisation signals as well as the mechanism to indicate the start and the end of the message transmission. As the method already uses beacon frames for bit synchronisation, the *Timestamp* field that is present in the beacon frame can be additionally used for message synchronisation. The Beacon frame does not have to originate from an access point that stations are connected to. The Beacon can be transmitted by any access point that operates on the same radio channel the stations are operating at.

The *Timestamp* field contains information about the time, in microseconds, that has elapsed since the access point was switched on. In a typical configuration, this field increases by about $10^{5}$ each time a beacon frame is transmitted (which is equivalent to the beacon frame interval of 0.1 s). If the value of the *Timestamp* field is divided by, for example, $SM=10^{7}$, we obtain a remainder that is reset to zero every 10 s. The remainder of a given *Timestamp* field being smaller than that of the previous *Timestamp* field (’counter rollover’) can be used as an indicator of the beginning of message transmission. With a divisor of $SM=10^{7}$, a message of a maximum of 100 bits can be sent approximately every 10 s. Other maximum message lengths are possible by using different divisors for message synchronisation, but its value has to be known for all participating nodes before the covert transmission begins. Since each station should receive the Beacon frames sent by the access point, this solution is highly reliable for synchronisation purposes.

Another method to synchronise covert message transmission is also possible and can involve an event that does not occur periodically. Such an event may, for example, be a specific type of frame from a predetermined station (e.g., an ARP frame sent by station A), etc. Such a synchronisation method enables the transmission to be started at any time and renders the covert transmission more challenging to detect because it does not begin at predetermined moments. On the other hand, one must provide such a periodic synchronisation occurrence. That complicates the implementation and introduces abnormal and potentially detectable network behaviour.

### 3.4. Variants of the StegoFrameOrder Method

The StegoFrameOrder method allows for many modifications by using a variety of parameters. One possible parameter is the type of chosen synchronisation method. When the timing depends on the access point, the SM value is an important parameter.

**Figure 4:**
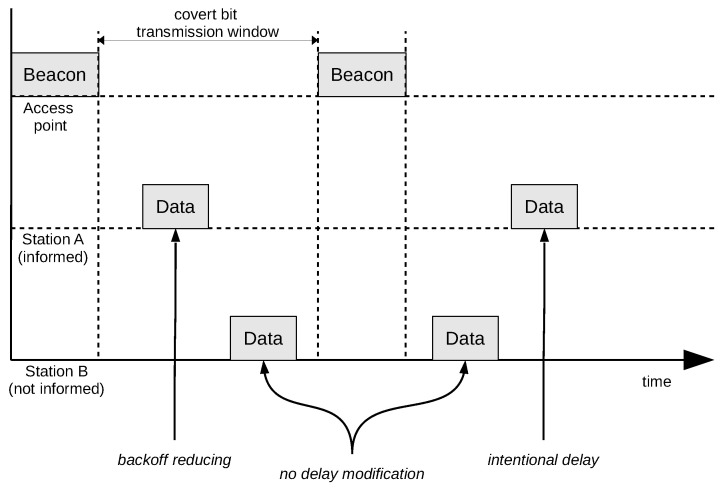
One-informed variant of the SFO method.

The SFO method can operate as two variants: with both (A and B) stations informed about the message to be transmitted (both informed, Figure 3), and with only one station (station A) informed. In the One-Informed case, the second station (station B) is transmitting regular network traffic with predictable frame frequency (Figure 4).

In the Two-informed scenario, the information being sent has to be known for both participants. In such a case, both nodes are cooperating in sending the covert message.

In the One-informed scenario, only one node is malicious and has to consider and exploit well-behaving network traffic from other nodes. Such transmission is more difficult to organise, as it has more conditions needed to operate.

The Both-informed variant is easier to implement because it requires only a mechanism for delaying the frame being sent (e.g., by storing it in the buffer until the frame from the other station has been received). The need to store covert information at both terminals can be cumbersome. For this reason, the One-informed variant is more robust as it stores covert information only on the informed station.

In this variant, the uninformed station B is used in the transmission involuntarily whenever it sends a large amount of data, for example, during file transfer. In this variant, an informed station A must be able to modify and shorten the backoff time to send messages ahead of the uninformed station B. The One-informed variant has the disadvantage that there is no guarantee that it can send a frame in the intended order. Station B must also transmit at least one frame in each beacon interval, which can be problematic if the station is beyond control. This difficulty can cause increased BER or reduced bandwidth of the covert channel.

Variants of the method that allows for the transmission of more than one bit of covert information during the beacon frame interval are also possible. Covert information can be embedded in the relative order of frames transmitted by more than two stations. The information capacity of channels in which multiple stations participate in the covert transmission is related to the number of possible frame sequences from all informed stations. The information capacity can be calculated from the permutation number of frame sequences using Formula (1):

(1) $$C=log_{2}\left(N!\right)$$

where *N* is the number of informed stations participating in covert transmission: for example, by using three stations, it becomes possible to send *C* = 2.58 bits of covert information and *C* = 4.58 bits of covert information between consecutive beacon frames with four stations.

**Figure 5:**
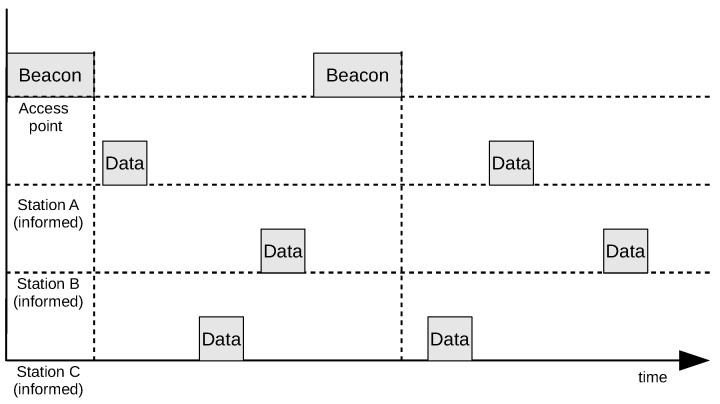
Variant of the SFO method with three informed stations.

However, such a relatively high information capacity implies a complex manner of synchronising stations and induces the need to inform multiple stations about the covert message being sent. The example of covert data transmission using three informed stations is shown in Figure 5. During the first beacon interval, the sequence of transmitted frames is ACB; during the second, it is CAB. Since the number of permutations of three elements is six, it is possible to represent one of six possible states during the beacon interval. Thus, 2.58 bits of covert data are transmitted per interval.

### 3.5. Covert Data Extraction Method

As for the transmission of secret information, there are several methods to receive and extract it. A primary method for this is derived from the nature of the IEEE 802.11 network and takes advantage of the fact that each station in a given network receives frames sent by other stations. In this method, named Station-detection, a station is connected to a wireless network and can also participate in transmission. It listens to frames from all stations and creates an order table of stations that have transmitted frames. Based on the analysis of this order, it extracts covert information.

**Figure 6:**
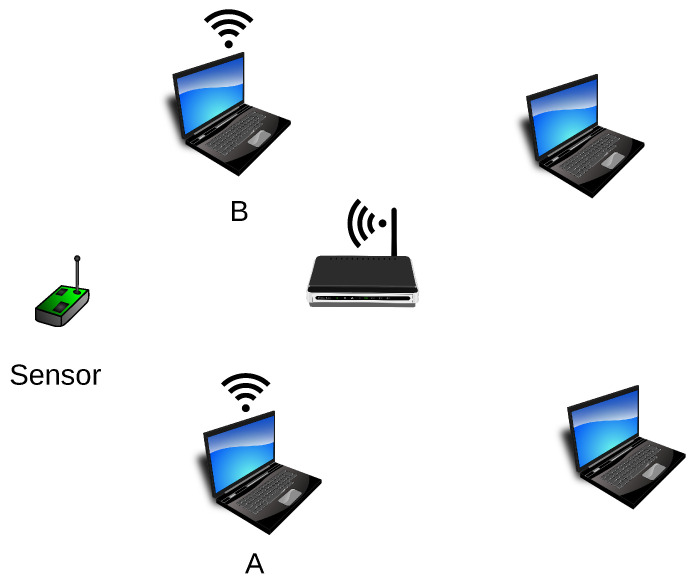
The Sensor-detection method (A and B—stations engaged in covert transmission).

Another method, named sensor-detection, involves the use of a sensor, i.e., a specialised device intended only to receive hidden information (Figure 6). Such a sensor is placed within the range of stations that transmit covert information and detects their sequence. The sensor does not need to be an active device (in the meaning of active transmission of frames) from the network’s point of view in which covert transmission occurs. This solution has undoubted advantages: The device receiving the covert information can be very small, difficult to detect, and can operate on battery power because there is no need to analyse the transmitted packets deeply. It is sufficient for the sensor to analyse the link layer of the received frames only.

An essential feature of this method of sending covert information is its low sensitivity to the activity of other stations operating on the same radio channel. Both the transmitting and the receiving sides ignore frames transmitted by non-participating stations. The transmission of a frame by any uninformed station does not disturb the relative order of frames originating from informed stations A and B.

### 3.6. Bandwidth

The bandwidth provided by the method that uses the synchronisation of the beacon frame interval can be calculated from the following formula:

(2) $$B=\frac{C}{T_{BI}\cdot 1024\;\mu s}$$

where *C* is the number of bits transmitted at once, calculated using Formula (1), and $T_{BI}$ is the beacon interval expressed in time units (TU = 1024 µs) defined by the IEEE 802.11 standard.

Assuming that the method allows for the transmission of one bit of covert information between beacon frames and that the beacon interval is 100 TU, the bandwidth of the hidden channel can be determined as follows.

(3) $$B=\frac{1\;b}{100\cdot 1024\;\mu s}=9.76\;\frac{b}{s}$$

## 4. Implementation

The StegoFrameOrder method has been implemented on an experimental setup with two basic office computers equipped with wireless network cards. For the IEEE 802.11n network operating on a 2.4 GHz band, the setup used USB cards with Atheros AR9271 chipset as their driver offers the possibility to work in station and monitor mode simultaneously. For the IEEE 802.11ac network, the setup used a set of cards with Ralink chipsets. The computers were controlled by the Debian GNU/Linux 10 operating system. The UniFi Mesh AC Pro device was used for the access point, as it supports 2.4 GHz and 5 GHz bands and both IEEE 802.11ac and IEEE 802.11n standards.

### 4.1. Both-Informed Variant

**Figure 7:**
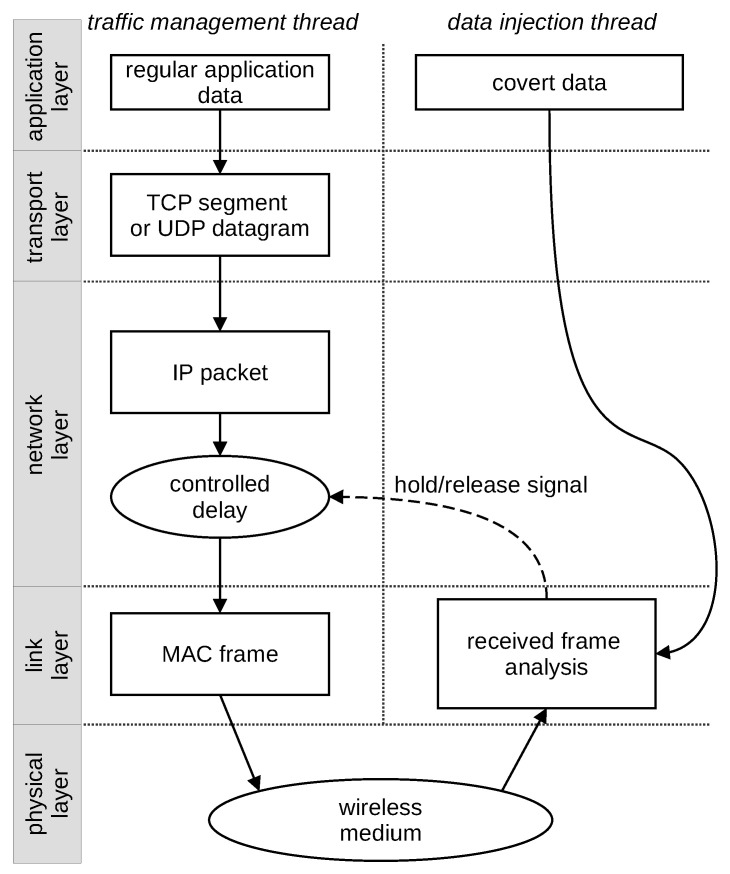
Multithreaded application for covert transmission control working scheme.

The StegoFrameOrder method was implemented in a Both-informed variant with synchronisation using the beacon frames. With this configuration, only a deliberate frame delay was required. This delay was induced by using the *libnetfilter_queue* library by a two-thread application running on each station. One thread was responsible for listening to radio transmission: detecting the transmission start and signalling other stations’ transmission. This thread required the wireless network card to work in monitor mode. The second thread was responsible for holding and releasing the frames in the buffer (Figure 7). Since the *libnetfilter_queue* mechanism was used, it was possible to delay frames in the network layer of the implementation. Covert data were received from the application layer by using a custom interface between the kernel and the user spaces in Linux. The software was written entirely in the C programming language and used open-source libraries only (*libnetfilter_queue* and *libpcap*).

### 4.2. One-Informed Variant

For the One-informed variant, the exact mechanisms and software were used, but the application was running only on one station. The second station’s requirements were as follows: working on the same BSSID and transmitting at least one data frame during every Beacon interval. The informed station was not specially prepared for the One-informed variant. There was no modification of the parameters related to medium access made.

### 4.3. Hardware and Software Challenges

The IEEE 802.11n (2.4 GHz) implementation used only one wireless USB card with Atheros AR9271 chipset per computer (the transmitting stations and a covert data receiver). The ath9k driver, responsible for controlling these cards in the Linux kernel, enables the possibility to work simultaneously in two modes: as a station connected to the access point and as a monitor intercepting frames transmitted by other stations operating on the same radio channel.

For the IEEE 802.11ac (5 GHz) implementation, we had to use a specific configuration for every transmitting station and the covert data receiver. From the set of different wireless network cards, we were not able to find one that could work in both monitor and station modes simultaneously and not drop the received frames. We were forced to use two different wireless cards on every transmitting station—one working in a station mode and the other working only as a monitor. We have chosen PCI-e cards with Ralink RTL8811au chipset for the station mode cards, which has a MIMO configuration of 1×1:1 (one Rx antenna, one Tx antenna, and one stream). For the monitor cards, we have used USB cards with Ralink RTL8812au (2×2:2 MIMO configuration). In the covert data receiver, we used a USB card with Ralink RTL8814AU (4×4:3 MIMO configuration). The same set of cards was selected after many attempts with different chipsets from vendors such as Ralink, Intel, and Qualcomm Atheros.

## 5. Experiments

The experiments were performed using the environment described in the implementation section.

**Figure 8:**
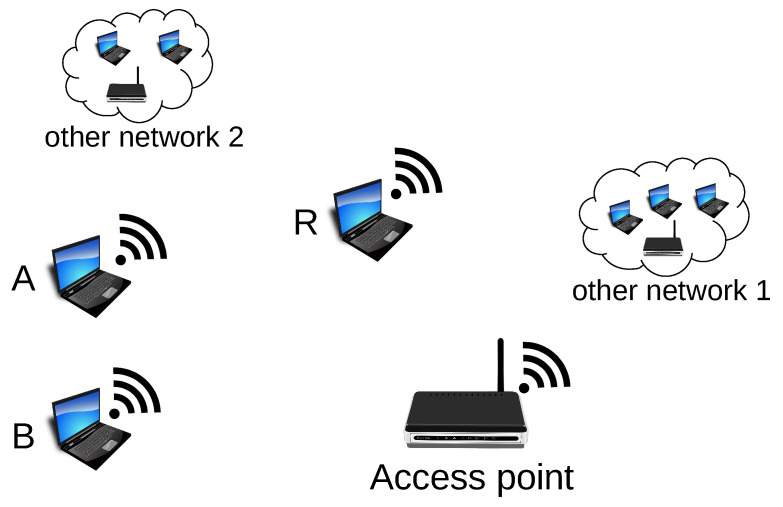
The stations used for the experiments in Both-informed variant (A and B—stations transmitting covert information; R—covert information receiver).

The first experiment was performed on the 2.4 GHz band with IEEE 802.11n devices. Since that band is widely used, the random operation of other network nodes and access points on the same radio channel was present during the experiment. The conditions were far from ideal as five independent stations and two access points were operating on the same radio channel. Such environment simulates the Wi-Fi wireless network’s actual, real-life conditions well. This experiment assumed that two stations transmit covert information simultaneously. The Both-informed stations were A and B (Figure 8). The third (R) microcomputer was introduced as a passive receiver of the covert messages (the sensor).

**Figure 9:**
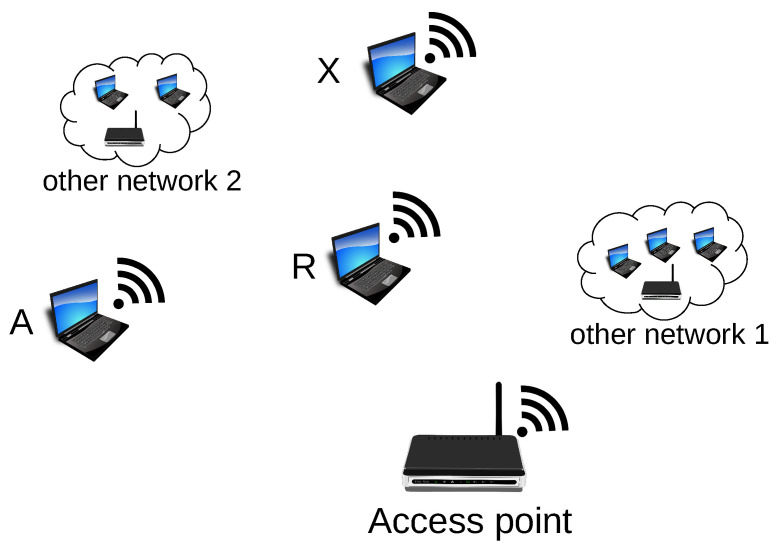
The experiment setup for “One-informed” variant. A—station transmitting covert information; X—the non-informed station; R—covert information receiver (sensor).

The second experiment was also performed in the IEEE 802.11n network operating on a 2.4 GHz band and served for evaluation of the “One-informed” scenario. The setup is shown in Figure 9.

The third experiment was performed in the IEEE 802.11ac network operating on a 5 GHz band with Both-informed stations. The experimental setup was almost identical to the one used in the first experiment. The only difference was network cards supporting IEEE 802.11ac standard and broader radio spectrum.

The One-informed variant performed in the IEEE 802.11ac network has not been tested, as the results of preliminary experiments were not satisfying.

The wireless networks used for all experiments were using the WPA2-PSK protocol for the security of transmitted data. The WPA2-PSK was chosen as it is currently the most popular security mechanism in small wireless networks.

Since only two stations are used to transmit and the Beacon frames are used as the source of synchronisation signal during the experiments, we achieved a covert data bandwidth of $C=10\frac{b}{s}$. The message transmission window was about 6 s long, which allowed us to transmit 32-bit long messages with some extra time that could be used if any of the Beacons originating from the access point were missed or corrupted.

### 5.1. Bit Error Rate and Packet Error Rate

The next step was to perform covert transmission in order to measure bit error rate (BER) and packet error rate (PER). We define the packet error rate as the percentage of messages received with at least one error bit:

(4) $$PER=\frac{N_{e}}{N}\cdot 100\%$$

where $N_{e}$ is the number of messages received with errors, and *N* is the number of all transmitted messages.

### 5.2. Reference Network Operation

We needed to establish the parameters of the reference network, such as the order of frames, from different network nodes in an unmodified network. The reference parameters were the base parameters used to assess the proposed method. Two unmodified wireless network terminals sent ICMP echo request packets, each of which had a size of 84 bytes, to a local access point at a rate of 20 packets per second. The command used for this was as follows.

# ping -i 0.05 192.168.1.1

Thanks to this, the most crucial assumption was satisfied: at least one frame from each station was transmitted between consecutive beacon frames. After 10 min of observation (approximately 5800 beacon frames), the order of frames transmitted by stations A and B was checked during every beacon interval. The frame from station A appeared before the one from station B in 49.98% of cases, and station B transmitted a frame before station A in 50.02% of cases. Therefore, the unmodified network showed near-perfect randomness in terms of the order of frames in intervals between beacon frames.

### 5.3. Both-Informed Variant on IEEE 802.11n Network

We have transmitted 4000 messages of 8, 16, 24, and 32-bit length (1000 messages of every length) during the experiment. On the receiving end (station R), all received messages were compared with the expected ones. Then, the BER and PER values were calculated. Stations A and B were configured to transmit two data frames for every Beacon interval.

**Figure 10:**
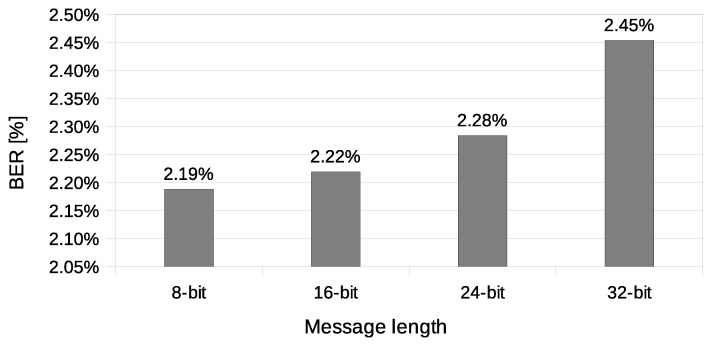
Measurement of the bit error rate (IEEE 802.11n; Both-informed).

The calculated BER values (Figure 10) were between 2.19% and 2.45%. These values are far from ideal, but it is important to note that the experiment was performed in an empirical scenario, with other wireless networks operating on the same radio channel interfering with its operation. It is still possible to send messages reliably with such relatively high BER value by using adequate redundancy (error correction codes such as BCH or Reed–Solomon’s code) [38,39] or message repetition in exchange for usable bandwidth. The goal of the experiments is to present the parameters of the proposed covert channel, and the experiments related to hidden data preparation (such as adding error correction codes) exceed the scope of the paper.

**Figure 11:**
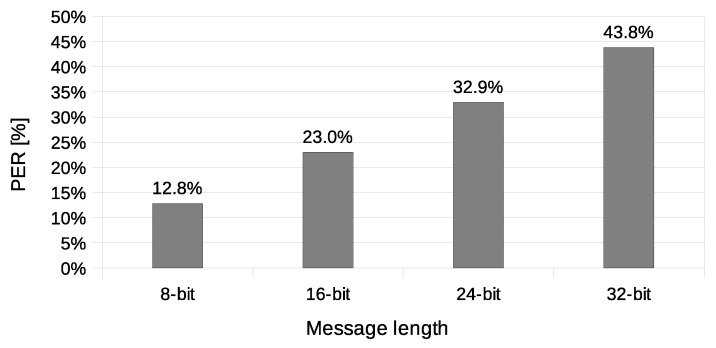
The packet error rate values (IEEE 802.11n, Both-informed).

**Figure 12:**
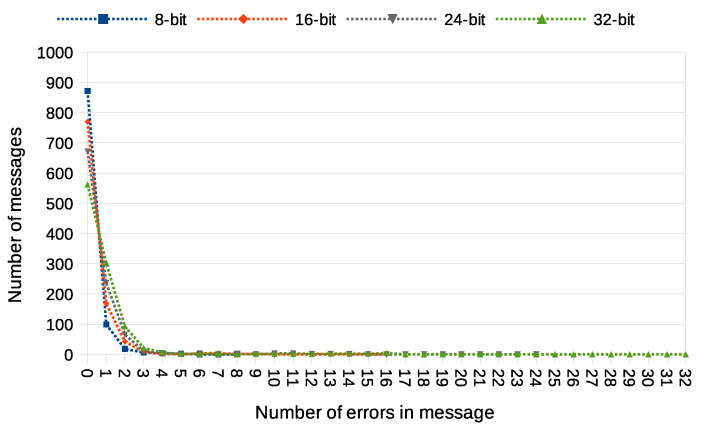
Numbers of messages received with different number of error bits (IEEE 802.11n; Both-informed).

**Figure 13:**
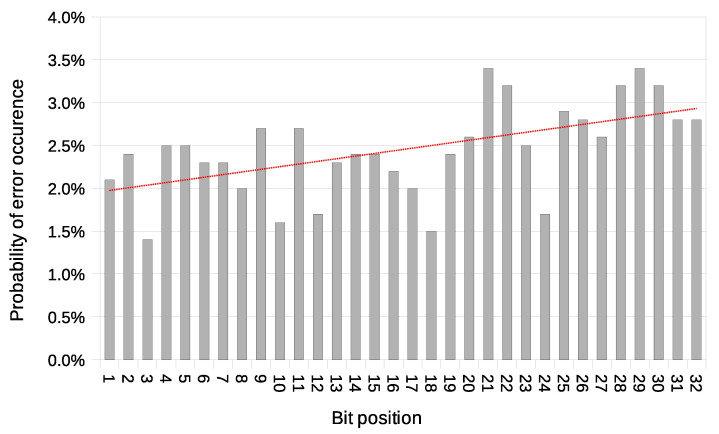
Probability of error occurrence as a function of bit position with a trend line (IEEE 802.11n; Both-informed).

Another used transmission quality metric is the packet error rate (PER) defined in Equation (4). The results of the packet error rate obtained in the experiment are shown in Figure 11. The 12.8% of 8-bit long messages and 43.8% 32-bit long messages were broken—with at least one bit altered (Figure 12). The shortest covert messages had the highest probability of being received as correct because of the lowest probability of desynchronisation resulting from Beacon frame ommision in one of the stations. If one of the stations does not receive the Beacon frame during the transmission window, it will desynchronise with the other station and send *n*-th bit when the other station sends the n+1 bit. If this happens, the received message will be broken from this erroneous bit until the end of the message. Due to this problem, it is more reliable to form and send shorter messages. The probability of error occurrence as a function of bit position is shown in Figure 13. One can observe the dependence between bit position and the probability of error occurrence. That phenomenon is most probably connected to non-ideal beacon time tracking that causes slow desynchronisation of the stations.

In order to fully understand these PER values, we also need to examine how many errors have been received in particular messages. This is shown in Figure 12. It is noticeable that most of the messages were received without errors or with a minimal number of errors. The number of the longest 32-bit messages received with at most one error bit is 864, which is an outstanding result compared to most steganographic methods of data transmission.

### 5.4. One-Informed Variant on IEEE 802.11n Network

**Figure 14:**
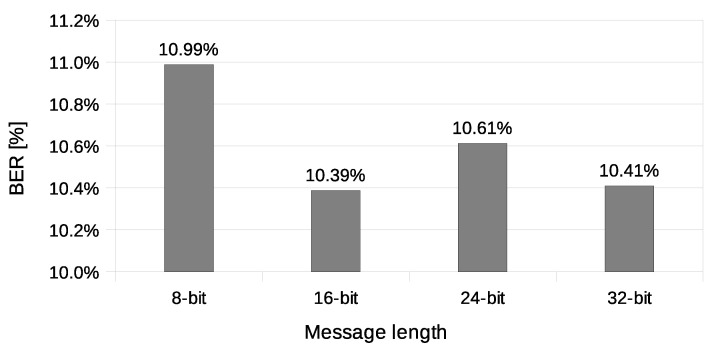
Measurement of the bit error rate (IEEE 802.11n; One-informed).

**Figure 15:**
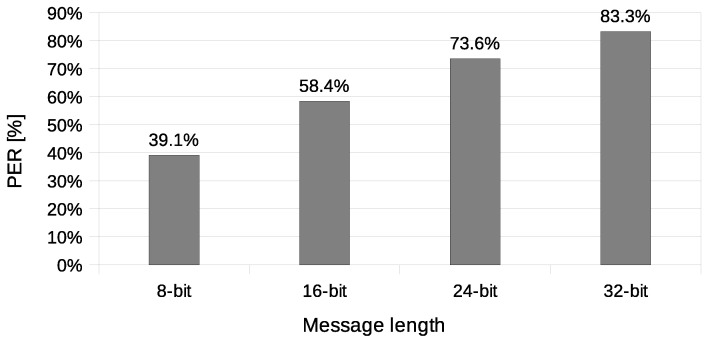
Measurement of the packet error rate (IEEE 802.11n; One-informed).

**Figure 16:**
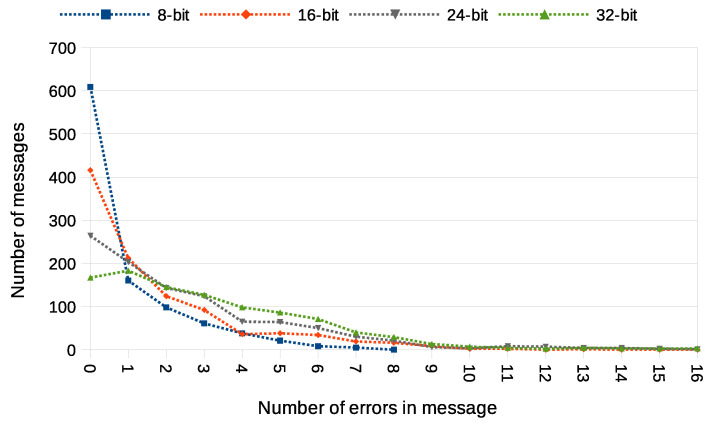
Numbers of messages received with different number of error bits (IEEE 802.11n; One-informed).

**Figure 17:**
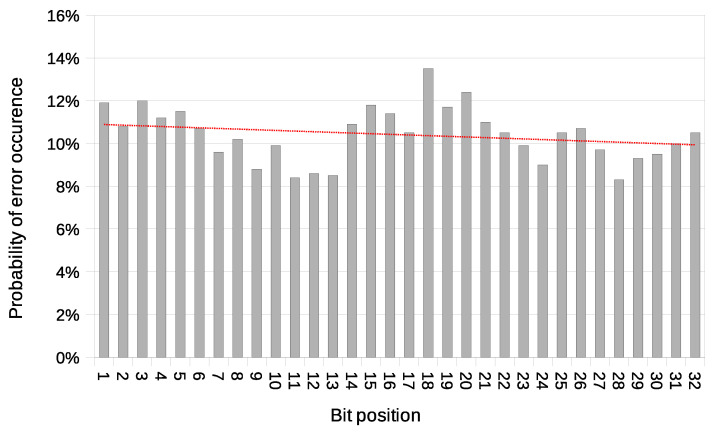
Probability of error occurrence as a function of bit position with a trend line (IEEE 802.11n; One-informed).

The experiment have included transmission of 1000 messages of each 8, 16, 24, and 32-bit length during this experiment. One station (A) was designated as the transmitter, and the other station (X) was sending data frames without any modification. Station A was configured to send four data frames for every beacon interval. Station X was configured to send one data frame for every beacon interval and did not have any knowledge of transmitted covert data. Results of this experiments are shown in Figure 14, Figure 15, Figure 16 and Figure 17.

The value of BER is about 10.5%, which is a significant value for the communication channel. It is essential to understand that the SFO method in the One-informed variant is working in an uncontrolled environment. The PER value for the shortest messages is relatively low. With error correction codes or message repetition, it is possible to achieve acceptable parameters of a covert channel constructed in that manner.

In this variant, the issue of possible desynchronisation between transmitting stations does not exist. Thanks to this, the BER value does not grow with message length. This characteristic is an advantage over the Both-informed variant. The probability of an error occurrence as a function of bit position is shown in Figure 17.

### 5.5. Both-Informed Variant on IEEE 802.11ac Network

**Figure 18:**
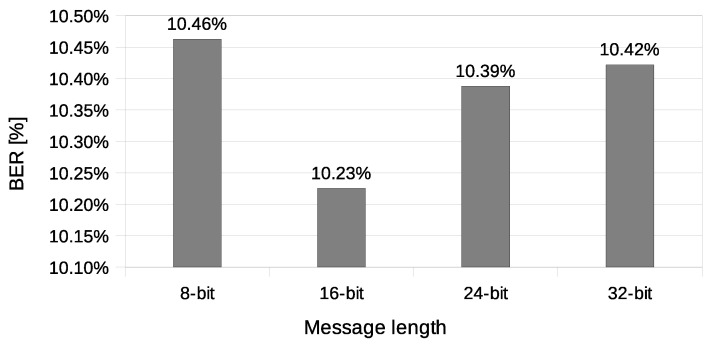
Measurement of the bit error rate (IEEE 802.11ac; Both-informed).

**Figure 19:**
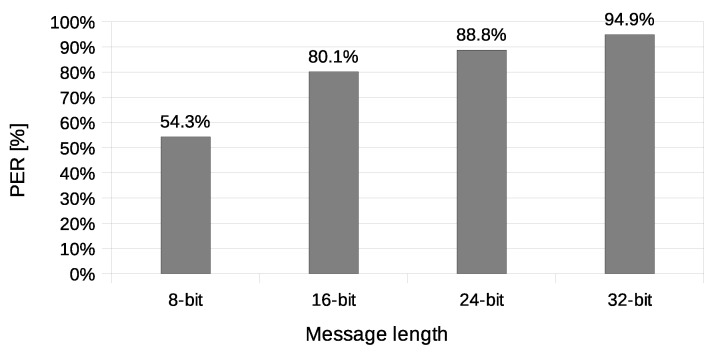
Measurement of the packet error rate (IEEE 802.11ac; Both-informed).

**Figure 20:**
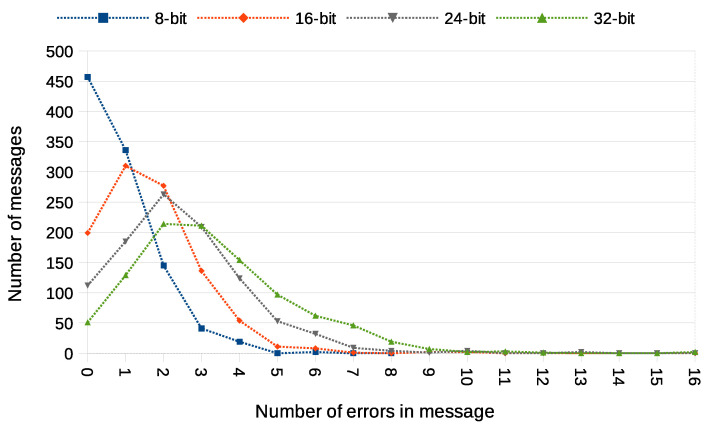
Numbers of messages received with different number of error bits (IEEE 802.11ac; Both-informed).

In the experiment with the Both-informed variant in a network working in IEEE 802.11ac standard, the total number of 4000 messages were transmitted—1000 of every length including 8, 16, 24, and 32-bit. Both transmitting stations were configured to send 20 data frames for every beacon interval. The results are shown in Figure 18, Figure 19 and Figure 20. The values of BER are similar to the values of BER in One-informed experiment results, but the PER is significantly larger.

**Figure 21:**
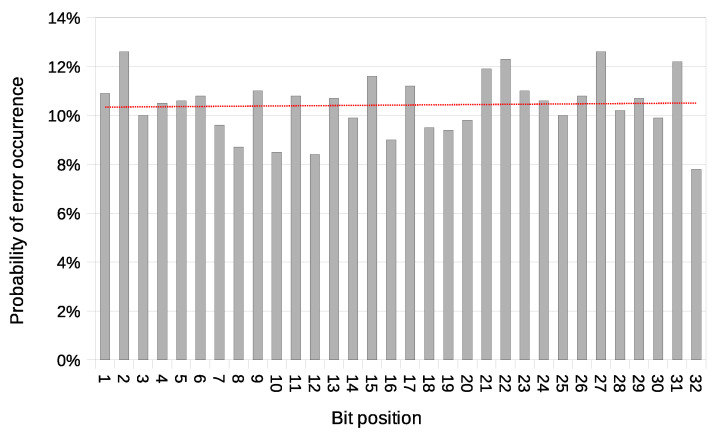
Probability of error occurrence as a function of bit position with a trend line (IEEE 802.11ac; Both-informed).

Relatively high error rates are attributed to the non-optimal experimental setup where there was a need to use two separate wireless cards. Two separate cards were needed because all 802.11ac cards that we were able to test did not allow simultaneous operation in monitor mode and client mode. One card working in client mode was transmitting frames controlled by the algorithm manner, while the second one was intercepting traffic when working in monitor mode. Since the intercepting card (working in the monitor mode) was connected via USB bus, the information about the occurrence of the data frame originating from the second informed station was received by the controlling application with a significant delay. It is the main disadvantage of the solution we had to use. The probability of an error occurrence as a function of the bit position in such setup is shown in Figure 21.

The BER value of about 10% can be made smaller when the transmitted data are prepared with an error correction code.

## 6. Conclusions

The method described in the paper makes it possible to establish a covert transmission channel in which properties are sufficient to present data or cryptography key leakage threat. Method performance was verified on the IEEE 802.11 wireless network. The implementation of the method yielded a relatively low bit error rate in real-life conditions that can be overcome easily with error correction techniques. It was also resistant to the presence of other non-informed stations operating on the same radio channel.

The SFO method advantages include the possibility of wide parametrisation, simple software implementation of both the covert information embedder and the extractor, and a relatively large bandwidth compared to other methods of steganography. Moreover, the proposed method does not compromise the receiver of covert information, which can be completely passive. This feature makes it more difficult to detect the station receiving covert data. The method does not introduce protocol overhead as it uses only frames that are intended to transmit and conveys regular data.

The major disadvantage of the SFO method is the requirement for all stations involved in covert transmission and the receiver to be placed in the distance, allowing them to receive frames from all involved stations. It should also be noted that the informed stations must generate at least one frame of regular data for every synchronisation window (Figure 3) to enable the method to work.

A statistical analysis of the sequences of frame sources used for covert transmission shows that the distribution of different frame sequences is unequal. This statistical anomaly results from using a direct encoding scheme and may result in the detection of the covert transmission through statistical steganalysis. However, if the covert data to be transmitted are prepared in a manner that they have an equal number of “0” and “1” bits (for example, by using 8b/10b encoding), it becomes difficult to detect this type of covert transmission. In this manner, it is possible to render the covert transmission imperceptible in exchange for usable bandwidth.

There are methods to improve the proposed method. A non-zero bit error rate problem can be solved by applying error detection and correction codes in exchange for smaller usable bandwidth. It is also possible to dynamically encode information by sequencing frames carrying covert information by using, for instance, the value of the Timestamp field of the beacon frame as the indicator of the beginning of transmission.

The proposed method can be adapted to different communication protocols that use a non-deterministic medium access algorithm. Different methods of synchronisation can significantly change the usable bandwidth of implementation. A very significant attribute of the SFO method is the possibility to implement it in real-life scenarios with the usage of off-the-shelf devices. This distinguishes the SFO method from other methods designed for wireless networks and published in the literature.

The bandwidth of the proposed method is adequate for the typical applications of steganography. For example, by using the presented method, it is possible to leak a 256-bit long cryptographic key to any passive node within the range of the network in less than 30 s. The demonstration of such a communication channel can contribute to more extensive security research regarding various media access protocols and potential security abuse mechanisms. Security auditing agencies should be interested in the development of a more extensive statistical analysis of network behaviour, especially in a time domain and check for statistical anomalies in frame intervals of individual nodes and statistical dependencies in sets of networked nodes. Thanks to the constantly growing number of ’Internet of Things’ devices that exploit wireless networks, it is an important topic. Most of these devices have closed-source firmware in which similar steganographic methods can be implemented and leak confidential or private information. For the sake of security, the importance of open-source software and open-hardware devices should increase. The paper shows that a relatively fast covert channel (the bandwidth of 10 bits per second is a significant value for covert channels) can be easily created and implemented in off-the-shelf devices only by using programming skills without changes to hardware.

## Data Availability

No data available.

## References

[1] Cox I., Miller M., Bloom J., Fridrich J., Kalker T. (2007). Digital Watermarking and Steganography.

[2] Mishra R., Bhanodiya P. A review on steganography and cryptography. Proceedings of the 2015 International Conference on Advances in Computer Engineering and Applications.

[3] Lubacz J., Mazurczyk W., Szczypiorski K. (2014). Principles and overview of network steganography. IEEE Commun. Mag..

[4] Bhunia S., Hsiao M.S., Banga M., Narasimhan S. (2014). Hardware Trojan Attacks: Threat Analysis and Countermeasures. Proc. IEEE.

[5] Guri M. (2021). Magneto: Covert channel between air-gapped systems and nearby smartphones via cpu-generated magnetic fields. Future Gener. Comput. Syst..

[6] Guri M. (2021). Exfiltrating data from air-gapped computers via ViBrAtIoNs. Future Gener. Comput. Syst..

[7] Cabaj K., Caviglione L., Mazurczyk W., Wendzel S., Woodward A., Zander S. (2018). The new threats of information hiding: The road ahead. IT Prof..

[8] Zielińska E., Mazurczyk W., Szczypiorski K. (2014). Trends in steganography. Commun. ACM.

[9] Wendzel S., Mazurczyk W., Caviglione L., Meier M. (2014). Hidden and uncontrolled–on the emergence of network steganographic threats. ISSE 2014 Securing Electronic Business Processes.

[10] Tanwar R., Malhotra S., Singh K. (2019). Future of Data Hiding: A Walk Through Conventional to Network Steganography. International Conference on Recent Developments in Science, Engineering and Technology.

[11] Venkatraman S., Abraham A., Paprzycki M. Significance of steganography on data security. Proceedings of the International Conference on Information Technology: Coding and Computing, 2004, Proceedings, ITCC 2004.

[12] Doshi R., Jain P., Gupta L. (2012). Steganography and its Applications in Security. Int. J. Mod. Eng. Res. (IJMER).

[13] Hassan W.H., binti Mohamad Noor M. (2019). Current research on Internet of Things (IoT) security: A survey. Comput. Netw..

[14] Lv Z., Qiao L., Kumar Singh A., Wang Q. (2021). AI-empowered IoT security for smart cities. ACM Trans. Internet Technol..

[15] Alladi T., Chamola V., Sikdar B., Choo K.K.R. (2020). Consumer IoT: Security vulnerability case studies and solutions. IEEE Consum. Electron. Mag..

[16] Xu L., Zhou X., Tao Y., Liu L., Yu X., Kumar N. (2021). Intelligent Security Performance Prediction for IoT-Enabled Healthcare Networks Using Improved CNN. IEEE Trans. Ind. Inform..

[17] Amanullah M.A., Habeeb R.A.A., Nasaruddin F.H., Gani A., Ahmed E., Nainar A.S.M., Akim N.M., Imran M. (2020). Deep learning and big data technologies for IoT security. Comput. Commun..

[18] (2017). McAfee Labs Threats Report. Http://mcafee.ly/2sXowrq.

[19] Securelist Steganography in Contemporary Cyberattacks. https://securelist.com/steganography-in-contemporary-cyberattacks/79276/.

[20] Wendzel S., Zander S., Fechner B., Herdin C. (2015). Pattern-based survey and categorization of network covert channel techniques. ACM Comput. Surv. (CSUR).

[21] Qiao G., Zhao Y., Liu S., Bilal M. (2017). Dolphin sounds-inspired covert underwater acoustic communication and micro-modem. Sensors.

[22] Tondwalkar A., Vinayakray-Jani P. (2016). Secure localisation of wireless devices with application to sensor networks using steganography. Procedia Comput. Sci..

[23] Murdoch S.J., Lewis S. (2005). Embedding covert channels into TCP/IP. International Workshop on Information Hiding.

[24] Rowland C.H. Covert Channels in the TCP/IP Protocol Suite 1997. https://firstmonday.org/ojs/index.php/fm/article/download/528/449.

[25] Ahsan K., Kundur D. Practical data hiding in TCP/IP. Proceedings of the Workshop on Multimedia Security at ACM Multimedia.

[26] Piotrowski Z., Sawicki K., Bednarczyk M., Gajewski P. New hidden and secure data transmission method proposal for military IEEE 802.11 networks. Proceedings of the 2010 Sixth International Conference on Intelligent Information Hiding and Multimedia Signal Processing.

[27] Jones E., Le Moigne O., Robert J.M. (2008). IP Time to Live (TTL) Field Used as a Covert Channel. U.S. Patent.

[28] Zander S., Armitage G., Branch P. (2006). Covert Channels in the IP Time to Live Field. https://researchrepository.murdoch.edu.au/id/eprint/35012/1/covert%20channels.pdf.

[29] Sawicki K., Piotrowski Z. The proposal of IEEE 802.11 network access point authentication mechanism using a covert channel. Proceedings of the 2012 19th International Conference on Microwaves, Radar & Wireless Communications.

[30] Frikha L., Trabelsi Z., El-Hajj W. Implementation of a Covert Channel in the 802.11 Header. Proceedings of the 2008 International Wireless Communications and Mobile Computing Conference.

[31] Nair A.S., Kumar A., Sur A., Nandi S. Length based network steganography using UDP protocol. Proceedings of the 2011 IEEE 3rd International Conference on Communication Software and Networks.

[32] Szczypiorski K. HICCUPS: Hidden communication system for corrupted networks. Proceedings of the International Multi-Conference on Advanced Computer Systems.

[33] Li S., Ephremides A. (2010). Covert channels in ad-hoc wireless networks. Ad Hoc Netw..

[34] Holloway R., Beyah R. Covert DCF: A DCF-based covert timing channel in 802.11 networks. Proceedings of the 2011 IEEE Eighth International Conference on Mobile Ad-Hoc and Sensor Systems.

[35] Sellke S.H., Wang C.C., Bagchi S., Shroff N. TCP/IP timing channels: Theory to implementation. Proceedings of the IEEE INFOCOM 2009.

[36] Tahmasbi F., Moghim N., Mahdavi M. (2016). Adaptive ternary timing covert channel in IEEE 802.11. Secur. Commun. Netw..

[37] Mohamed E.E., Mnaouer A.B., Barka E. PSCAN: A Port Scanning Network Covert Channel. Proceedings of the 2016 IEEE 41st Conference on Local Computer Networks (LCN).

[38] Berlekamp E.R. (1980). The technology of error-correcting codes. Proc. IEEE.

[39] Van Wonterghem J., Alloum A., Boutros J.J., Moeneclaey M. Performance comparison of short-length error-correcting codes. Proceedings of the 2016 Symposium on Communications and Vehicular Technologies (SCVT).

